# Pearls and Pitfalls in Diagnosing Non-Radiographic Axial Spondyloarthritis

**DOI:** 10.31138/mjr.33.1.109

**Published:** 2022-04-15

**Authors:** Styliani Tsiami, Xenofon Baraliakos

**Affiliations:** Rheumazentrum Ruhrgebiet Herne, Ruhr University Bochum, Germany

**Keywords:** nr-axSpA, MRI, stress-induced, degenerative

## Abstract

Although nr-axSpA is a distinct clinical entity with characteristic clinical and radiologic features, it is mimicked by a variety of other stress-induced, degenerative, infectious diseases or other conditions both clinically and radiologically, especially when it comes to the interpretation of imaging methods such as magnetic resonance imaging (MRI). Overall, the sensitivity and specificity of MRI in the diagnosis of nr-axSpA has limitations and must be interpreted in the context of the clinical picture. Furthermore, the interpretation of sacroiliac joint MRI is critical to avoid overdiagnosis as nr-axSpA because bone marrow oedema adjacent to the sacroiliac joint may also be frequently observed in people without axSpA such as post-partum women and athletes, even in the general population.

In this review article we present recent updates about the various disease entities and conditions that may mimic nr-axSpA and how to differentiate among them especially by imaging with MRI.

## INTRODUCTION

The Assessment of SpondyloArthritis international Society (ASAS) published in 2009 the ASAS classification criteria for axial (ax) spondyloarthritis (SpA),^1^ which has led to the distinction between the classical ankylosing spondylitis (AS) or radiographic axSpA and non-radiographic (nr) axSpA. ‘Non-radiographic’ is defined as the absence of definite radiographic sacroiliitis according to the modified New York criteria.^2^ Patients who present with a clinical picture suspicious for SpA but have not yet developed radiographic sacroiliitis, had been diagnosed earlier by rheumatologists as having “undifferentiated spondyloarthritis” (uSpA). These patients would be diagnosed today as having nr-ax-SpA based on magnetic resonance imaging (MRI), with signs of active sacroiliitis and concomitant clinical manifestations. In the last three years, there are increasing reports that the ASAS definition of a positive MRI, mainly based on the quantification of bone marrow oedema lesions suggestive of SpA, is not as specific as initially thought.^3–9^ The aim of this review is to describe and discuss the role of imaging for diagnosis of nr-axSpA and the various differential diagnoses that show similar imaging characteristics.

### Epidemiology of nr-axSpA

The prevalence of AS has been well studied and was found to be between 0.1 and 1.4%.^10,11^ In contrast, the epidemiology of nr-axSpA is still being investigated. A retrospective cohort study, based on the analysis of medical records from representative rheumatology practices in the United States, found after extrapolating the data to the national level that the U.S. prevalence of nr-axSpA according to ASAS criteria is 0.35% and similar to that for AS.^12^ Overall, except for the male:female ratio, no major differences in patient demographics have been reported between both SpA subgroups. AS shows a clear male dominance with a male-to-female ratio of up to 3:1.^13,14^ In contrast, nr-axSpA patients show little difference in the prevalence between males and females.^15,16^ Almost no differences were found regarding the mean age of the patients at presentation between subgroups.

### Clinical characteristics of nr-axSpA

Several cohort studies examined the demographic and clinical features of patients with nr-axSpA in comparison to AS patients, seeking conclusive evidence that both disorders represent a spectrum of the same disease.^17^ Patients with AS or with nr-axSpA may present with characteristic clinical features such as inflammatory back pain (IBP), with peripheral symptoms such as enthesitis or arthritis, and with extra-musculoskeletal manifestations such as anterior uveitis, psoriasis and chronic inflammatory bowel disease.^18,19^ Furthermore, many patients, especially those who are positive for human leucocyte antigen (HLA) B27, have a positive family history of SpA or related diseases.^20^ The observed dissimilarities between the nr-axSpA and AS cohorts included longer disease duration, higher degree of radiographic damage, and reduced spinal mobility in AS patients.

### Imaging of nr-axSpA

The diagnosis of nr-axSpA in the clinical setting can be challenging and advanced imaging has become essential for its recognition, as well as for the differential diagnosis. Because the disease affects sacroiliac joints (SIJ) in most patients, imaging of SIJ has a pivotal role for diagnosis of nr-axSpA,^21^ while the spine is less frequently involved.^22^

### Radiographs in nr-axSpA

Conventional radiography of the SIJ is recommended as the first imaging method to diagnose sacroiliac joint involvement as part of axSpA and to a further extent in its classification.^2,23^

**Figure 1.**
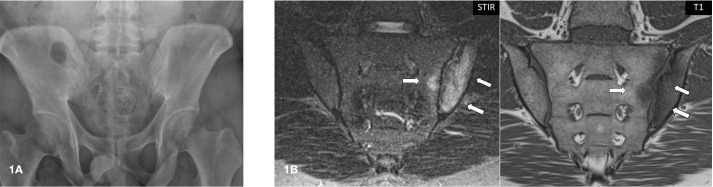
**(A)** Radiograph of the SIJ from a patient with nr-axSpA without pathological findings. **(B)** MRI of the same patient. Oblique coronal STIR and T1-weighted images. Pronounced bone marrow oedema on the left SIJ (white arrows), shown as hyperintense signal on STIR and hypointense signal on T1-weighted MR, especially at the iliac bone.

The term nr-axSpA, as mentioned before, is used for patients suffering from axSpA, but where the standard diagnosis, based on the presence of sacroiliitis on X-ray images, does not apply by the absence of radiographic changes.^2^ (**Figure 1A**). These patients are therefore regarded as ‘non-radiographic’. However, due to the complex anatomy of the SIJ, interpretation of these radiographs is often challenging. Indeed, a considerable inter-reader variation when evaluating conventional radiographs has repeatedly been reported even among experienced readers.^24^

### Magnetic resonance imaging in nr-axSpA

Normal or ambiguous radiographic results of SIJ examination in the context of a possible diagnosis of SpA require MRI investigation of the SIJ as the next step.^25^

While it may take up to 10 years for the first structural lesions to appear on pelvic radiography, MRI has the potential to detect inflammation at the very first manifestation of sacroiliitis. Moreover, MRI has shown the ability to also demonstrate inflammation-related structural SIJ lesions in 60–90 % of SpA patients already in the first 2–3 years after symptom onset.^26^

The following MRI sequences are useful for diagnosis and differential diagnosis of axSpA: a T2-weighted sequence with fat suppression (such as a short tau inversion recovery [STIR] sequence) for detection of active inflammatory changes (bone marrow oedema [BMO]) and a T1-weighted sequence for detection of post-inflammatory changes, such as erosions, sclerosis, ankyloses, and fatty lesions.

According to the ASAS criteria a positive scan is defined as one area of BMO on at least two consecutive slices or at least two areas of BMO on a single slice, while lesions such as capsulitis, enthesitis and synovitis should also be taken into account also.^27^ (**Figure 1B**).

The presence of structural damage without BMO is currently not sufficient according to ASAS, for classification to the disease. As mentioned before, more recent research reinforces the notion that inflammatory changes on an MRI, without suspicious changes in the conventional radiograph, should not be used in isolation to identify nr-axSpA.^7–13^ It is therefore important to mention that periarticular BMO signal can appear in patients with non-specific back pain,^4^ postpartum women,^5^ soldiers,^6^ runners,^5^ athletes,^7^ and even in the general population,^8,28^ emphasizing the importance of a proficient and expert reading of the MRI images for an accurate identification of BMO in the context of SpA diagnosis.

Common differential diagnoses for nr-axSpA are degenerative or mechanical problems (osteitis condensans, osteoarthritis of SIJ, accessory SIJs), while other differential diagnoses such as fractures and infectious sacroiliitis are less frequent but still possible.

#### Osteitis condensans ilii

**Figure 2.**
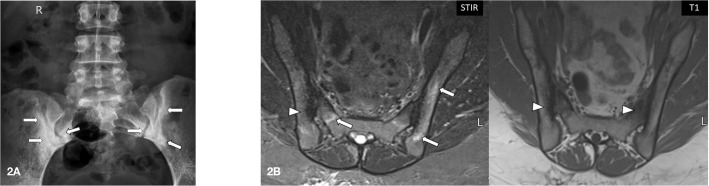
**(A)** Radiograph of the SIJ from a 25 yo female patient multipara with chronic lower backpain caused by OCI. Moderate paraarticular multisclerosis in the SIGs emphasized on both iliac sides without definite evidence of erosions or ankyloses. **(B)** MRI of the same patient. Oblique transversal STIR and T1-weighted images of the SIJ. Paraarticular bone marrow oedema, shown as hyperintense signal on STIR (white arrows) and paraarticular multisclerosis of the SIJ bilateral, sacrum and ilium side, without erosive changes, shown as hypointense signal on T1-weighted MR (white arrowheads).

Osteitis condensans ilii (OCI) is a condition that can present with chronic back pain and sometimes hip pain. In this case, probably mostly in the context of mechanical stress, bilateral sclerosis occurs in the area of the distal SIJ with a preference for the iliac bone. Such radiographic changes are frequently mimicking sacroiliitis such as the one found in nr-axSpA (**Figures 2A and 2B**). The hypothesis that previous pregnancies might be a precipitating factor was supported by the results of a case-control study of 35 patients identified with OCI over a 10-year period. All patients were female and reported previous pregnancies.^29^ SIJ stress tests showed higher SIJ pressure sensitivity in OCI patients compared with healthy controls. Number of pregnancies, birth weight, and back pain symptomatology did not differ between OCI patients and controls.

One recent prospective study compared patients with OCI and with axSpA, all of whom had been referred for possible axSpA.^30^ Almost all patients with OCI were women who reported frequent pregnancies (83%, mean number of 3 pregnancies). The OCI patients had an overall significantly lower prevalence of inflammatory back pain, and typical SpA features were less frequent than in axSpA, but also more frequent than in chronic back pain patients. A statistically significant difference compared to axSpA was only found for anterior uveitis. The age at onset of back pain was not different axSpA and OCI, and there were no differences in spinal mobility. Also, disease activity (Bath AS Disease Activity Index, BASDAI) and subjective degree of functional impairment (Bath ankylosing spondylitis functional index, BASFI) were comparable between the groups. Although 84% of axSpA patients were HLAB27+, 35% of the patients classified as OCI patients also carried this genetic trait (compared to 8% in the in the general population). An elevated CRP was found in about 40% of the axSpA and in only 7% of OCI patients. Overall, the results of these studies shown that a distinction between axSpA and OCI is difficult in daily practice.

### Osteoarthritis of sacroiliac joints

It has long been known that degeneration of SIJ is common and causes low back pain.^31^ Degenerative changes of SIJ are characteristic and bone sclerosis is the main feature, with SIJ space narrowing in only about 1/4 of the cases. Sclerosis is fairly limited to the anterior and middle portion of the joints, that often manifests as sharp and well demarcated and dense area, compared to the moderately dense and fuzzy edged sclerosis in inflammatory sacroiliitis.^32^

In a retrospective analysis of 281 MRI examinations performed for low back pain in 116 men and 165 women, mean age 44±15 years,^33^ sacroiliitis according to ASAS criteria was found in 71 examinations (25%), whereas degenerative changes were found in 11 patients (4%). These changes were defined as joint space narrowing, subchondral sclerosis, subchondral cysts, osteophytes and minute subchondral fat deposition with or without minor subchondral BMO. Patients with alternative diagnoses were older than patients with sacroiliitis (62 vs. 47 years of age, respectively); however, this difference was not statistically different. A similar study performed by Jans et al. reported slightly lower percentage of SIJ degenerative changes (3.6%).^34^

### Accessory sacroiliac joints

Accessory SIJs are an articulation between the medial aspect of the posterior superior iliac spine and the sacrum just lateral to the second dorsal sacral foramen. They may be congenital (diarthrodial joint) or more commonly acquired (fibrocartilaginous joint) in origin.^35^ The prevalence of accessory SIJs has been described in 13–18% of the general population, found bilaterally in 50% of affected persons.^36^

Young patients complain of chronic or recurrent low back pain, which makes this SIJ- anatomical variation a differential diagnosis of nr-axSpA.

Rafei et al. identified in their retrospective analysis of 157 MRI examinations of SIJ, the variation of accessory SIJ in 17 patients. This variation was best depicted on the axial images and was located at the level of the first or second sacral foramen. Signal intensity changes of the adjacent bone could be depicted in 11 patients, mostly of structural type (sclerosis of fatty deposition), but 4 patients also demonstrated oedematous changes.^37^

### Sacral stress fractures

**Figure 3.**
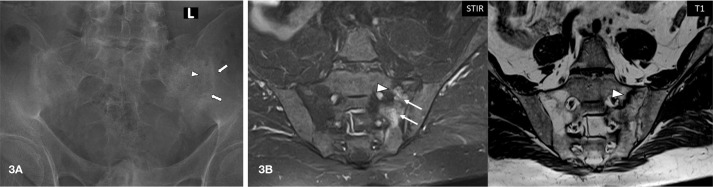
**(A)** Radiograph of the pelvis from a patient with low back pain and structural SIJ findings indicating post-inflammatory changes (arrows) and suspicion of fracture (arrowhead). **(B)** MRI of the same patient with unilateral sacral insufficiency fracture on the left SIJ. The oblique coronal T1-weighted image shows a typical hypointense fracture-line (arrowhead), with surrounding bone marrow oedema, shown as hyperintense signal on STIR (white arrows)

Sacral stress fractures may represent an underestimated cause of prolonged low back and pelvic pain both in athletically active young to middle-aged persons.^38,39^ Patients with undiagnosed sacral stress fractures are usually referred to radiographs of the pelvis and lumbar spine, which usually do not reveal the pathology. On account of a combination of low back pain and normal radiographs is the suspicion of nr-axSpA justified (**Figure 3A**). On MRI of the SIJ, BMO in the periphery of the sacrum, suggestive of a stress fracture, may be mistaken for the periarticular BMO of sacroiliitis. In this case, the fracture line should be looked for on both T1w as well as T2w sequences within the BMO.^40^ (**Figure 3B**)

### Infectious sacroiliitis

Infectious diseases also present an important differential diagnosis of axSpA, since a delayed diagnosis can lead to irreversible damages of the joint. Since mostly young people are affected by pyogenic sacroiliitis,^41,42^ by far its most common symptom is the deep-seated back pain^43^ infection of the SIJ may mimic especially nr-axSpA.

Staphylococcus aureus is the most frequent organism recovered from synovial or blood specimens in patients with infectious sacroiliitis, but streptococcus species, Escherichia coli, and salmonella species have also been reported.^44^

**Figure 4.**
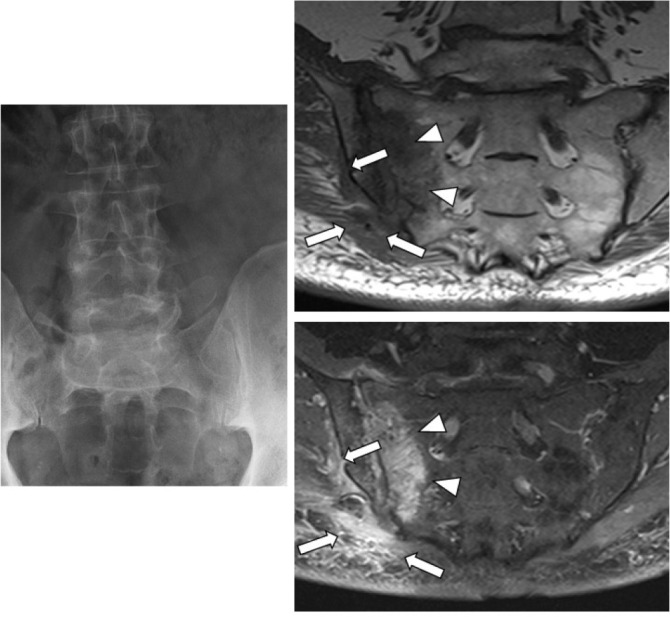
Radiograph and MRI of the SIJ of a 40 yo male patient with low back pain and elevated CRP. The radiograph shows no clear pathological findings. Oblique coronal MRI images after Gadolinium (Gd) application, showing hyperintense (inflammatory) signal periarticularly locates (arrowheads) but also in the gluteal muscle area (arrows), pointing towards a bacterial inflammation.

Especially important to mention is brucellosis, which is endemic to the Mediterranean area and the Middle East. Sacroiliitis from brucellosis has been reported in 0–72% of the patients in different series.^45,46^ SIJ involvement may occur together with spondylitis, and it might be hard to distinguish from other causes of sacroiliitis, like nr-axSpA as no distinct radiological findings are present in the first 2–3 weeks other than blurring, indistinctness of the subchondral osseous line and narrowing or widening of the interosseous space. MRI is the imaging modality of choice demonstrating intra-articular fluid, BMO with predominant periarticular involvement, especially during the early phase of the brucellosis.^47^ (**Figure 4**).

## CONCLUSION

We describe here the most common and challenging radiographic and clinical mimickers of nr-axSpA, based on imaging findings. Various studies have highlighted a relatively high prevalence of BMO on MRI of SIJs in healthy volunteers, which could even be categorized as having a false positive classification as defined by ASAS. In the future it will be important to define clearly which MRI finding is stress-induced, degenerative, infectious, and/or non-specific versus specific for axSpA.

## References

[1] Rudwaleit M Landewé R Van Der Heijde D Listing J Brandt J Braun J The development of Assessment of SpondyloArthritis international Society classification criteria for axial spondyloarthritis (part I): Classification of paper patients by expert opinion including uncertainty appraisal. Ann Rheum Dis 2009 Jun;68(6):770–6. 19297345 10.1136/ard.2009.108217

[2] Linden S Van Der Valkenburg HA Cats A. Evaluation of Diagnostic Criteria for Ankylosing Spondylitis. Arthritis Rheum 1984;27(4):361–8. 6231933 10.1002/art.1780270401

[3] De Bruin F Treyvaud MO Feydy A De Hooge M Pialat JB Dougados M Prevalence of degenerative changes and overlap with Spondyloarthritis-Associated lesions in the spine of patients from the DESIR cohort. RMD Open 2018;4(1). 10.1136/rmdopen-2018-000657 PMC6018874 29955382

[4] Arnbak B Grethe Jurik A Hørslev-Petersen K Hendricks O Hermansen LT Loft AG Associations between Spondyloarthritis Features and Magnetic Resonance Imaging Findings: A Cross-Sectional Analysis of 1,020 Patients with Persistent Low Back Pain. Arthritis Rheumatol 2016 Apr 1;68(4):892–900. 26681230 10.1002/art.39551

[5] de Winter J de Hooge M van de Sande M de Jong H van Hoeven L de Koning A Magnetic Resonance Imaging of the Sacroiliac Joints Indicating Sacroiliitis According to the Assessment of SpondyloArthritis international Society Definition in Healthy Individuals, Runners, and Women With Postpartum Back Pain. Arthritis Rheumatol 2018 Jul 1 ;70(7):1042–8. 29513924 10.1002/art.40475 PMC6032910

[6] Varkas G De Hooge M Renson T De Mits S Carron P Jacques P Effect of mechanical stress on magnetic resonance imaging of the sacroiliac joints: Assessment of military recruits by magnetic resonance imaging study. Rheumatol (United Kingdom) 2018 Mar 1;57(3):508–13. 10.1093/rheumatology/kex491 29253272

[7] Weber U Jurik AG Zejden A Larsen E Jørgensen SH Rufibach K Frequency and Anatomic Distribution of Magnetic Resonance Imaging Features in the Sacroiliac Joints of Young Athletes: Exploring “Background Noise” Toward a Data-Driven Definition of Sacroiliitis in Early Spondyloarthritis. Arthritis Rheumatol 2018 May 1;70(5):736–45. 29430880 10.1002/art.40429

[8] Baraliakos X Richter A Feldmann D Ott A Buelow R Schmidt CO Frequency of MRI changes suggestive of axial spondyloarthritis in the axial skeleton in a large population-based cohort of individuals aged <45 years. Ann Rheum Dis 2019;79(2). 10.1136/annrheumdis-2019-215553 31744822

[9] De Bruin F Ter Horst S Bloem HL Van den Berg R De Hooge M Van Gaalen F Prevalence of degenerative changes of the spine on magnetic resonance images and radiographs in patients aged 16–45 years with chronic back pain of short duration in the Spondyloarthritis Caught Early (SPACE) cohort. Rheumatol (United Kingdom) 2016 Jan 1;55(1):56–65. 10.1093/rheumatology/kev283 26275972

[10] Reveille JD Witter JP Weisman MH. Prevalence of axial spondylarthritis in the United States: Estimates from a cross-sectional Survey Vol. 64, Arthritis Care and Research. Arthritis Care Res (Hoboken);2012. p. 905–10. 22275150 10.1002/acr.21621 PMC4032290

[11] Costantino F Talpin A Said-Nahal R Goldberg M Henny J Chiocchia G Prevalence of spondyloarthritis in reference to HLA-B27 in the French population: Results of the GAZEL cohort. Ann Rheum Dis 2015 Apr 1 ;74(4):689–93. 24351517 10.1136/annrheumdis-2013-204436

[12] Strand V Rao SA Shillington AC Cifaldi MA McGuire M Ruderman EM. Prevalence of axial spondyloarthritis in united states rheumatology practices: Assessment of spondyloarthritis international society criteria versus rheumatology expert clinical diagnosis. Arthritis Care Res 2013 Aug ;65(8):1299–306. 10.1002/acr.21994 23436774

[13] Van Der Horst-Bruinsma IE Zack DJ Szumski A Koenig AS. Female patients with ankylosing spondylitis: Analysis of the impact of gender across treatment studies. Ann Rheum Dis 2013 Jul;72(7):1221–4. 23264358 10.1136/annrheumdis-2012-202431

[14] Gran JT Husby G. Ankylosing spondylitis in women. Semin Arthritis Rheum 1990;19(5):303–12. 2192459 10.1016/0049-0172(90)90053-i

[15] Sieper J Van Der Heijde D. Review: Nonradiographic axial spondyloarthritis: New definition of an old disease? Vol. 65, Arthritis and Rheumatism. Arthritis Rheum; 2013. p. 543–51. 23233285 10.1002/art.37803

[16] Gremese E Bernardi S Bonazza S Nowik M Peluso G Massara A Body weight, gender and response to TNF-α blockers in axial spondyloarthritis. Rheumatol (United Kingdom) 2014;53(5):875–81. 10.1093/rheumatology/ket433 24407233

[17] Baraliakos X Braun J. Non-radiographic axial spondyloarthritis and ankylosing spondylitis: What are the similarities and differences? Vol. 1, RMD Open. BMJ Publishing Group; 2015. 10.1136/rmdopen-2015-000053 PMC4632143 26557375

[18] Braun J Sieper J. Ankylosing spondylitis Vol. 369. Lancet. Elsevier B.V.; 2007. p. 1379–90. 17448825 10.1016/S0140-6736(07)60635-7

[19] Dougados M Baeten D. Spondyloarthritis. Vol. 377, The Lancet. Elsevier B.V.; 2011. p. 2127–37. 10.1016/S0140-6736(11)60071-8 21684383

[20] Braun J Baraliakos X. Imaging of axial spondyloarthritis including ankylosing spondylitis. Ann Rheum Dis 2011 2011 Mar;70 Suppl 1:i97–103. 21339229 10.1136/ard.2010.140541

[21] Sieper J Rudwaleit M Baraliakos X Brandt J Braun J Burgos-Vargas R The Assessment of SpondyloArthritis international Society (ASAS) handbook: A guide to assess spondyloarthritis. Ann Rheum Dis 2009 Jun;68(SUPPL. 2). 10.1136/ard.2008.104018 19433414

[22] Kiltz U Baraliakos X Karakostas P Igelmann M Kalthoff L Klink C Do patients with non-radiographic axial spondylarthritis differ from patients with ankylosing spondylitis? Arthritis Care Res 2012 Sep;64(9):1415–22. 10.1002/acr.21688 22505331

[23] Van Den Berg R De Hooge M Rudwaleit M Sieper J Van Gaalen F Reijnierse M ASAS modification of the Berlin algorithm for diagnosing axial spondyloarthritis: Results from the SPondyloArthritis Caught Early (SPACE)-cohort and from the Assessment of SpondyloArthritis international Society (ASAS)-cohort. Ann Rheum Dis 2013 Oct;72(10):1646–53. 23139266 10.1136/annrheumdis-2012-201884

[24] Van Den Berg R Lenczner G Feydy A Van Der Heijde D Reijnierse M Saraux A Agreement between clinical practice and trained central reading in reading of sacroiliac joints on plain pelvic radiographs: Results from the DESIR cohort. Arthritis Rheumatol 2014;66(9):2403–11. 24909765 10.1002/art.38738

[25] Mandl P Navarro-Compán V Terslev L Aegerter P Van Der Heijde D D’Agostino MA EULAR recommendations for the use of imaging in the diagnosis and management of spondyloarthritis in clinical practice Vol. 74, Annals of the Rheumatic Diseases. BMJ Publishing Group; 2015. p. 1327–39. 10.1136/annrheumdis-2014-206971 25837448

[26] Althoff CE Sieper J Song IH Haibel H Weiß A Diekhoff T Active inflammation and structural change in early active axial spondyloarthritis as detected by whole-body MRI. Ann Rheum Dis 2013 Jun;72(6):967–73. 22736088 10.1136/annrheumdis-2012-201545

[27] Lambert RGW Bakker PAC Van Der Heijde D Weber U Rudwaleit M Hermann KGA Defining active sacroiliitis on MRI for classification of axial spondyloarthritis: Update by the ASAS MRI working group. Ann Rheum Dis 2016 Nov 1;75(11):1958–63. 26768408 10.1136/annrheumdis-2015-208642

[28] Baraliakos X Richter A Feldmann D Ott A Buelow R Schmidt CO Which factors are associated with bone marrow oedema suspicious of axial spondyloarthritis as detected by MRI in the sacroiliac joints and the spine in the general population? Ann Rheum Dis 2021 Apr 1;80(4):469–74. 33239274 10.1136/annrheumdis-2020-218669

[29] Jenks K Meikle G Gray A Stebbings S. Osteitis condensans ilii: A significant association with sacroiliac joint tenderness in women. Int J Rheum Dis 2009;12(1):39–43. 20374315 10.1111/j.1756-185X.2009.01378.x

[30] Poddubnyy D Weineck H Diekhoff T Redeker I Gobejishvili N Llop M Clinical and imaging characteristics of osteitis condensans ilii as compared with axial spondyloarthritis. Revmatol 2020 Dec 1;59(12):3798–806. 10.1093/rheumatology/keaa175 32447391

[31] Resnick D Niwayama G Goergen TG. Degenerative disease of the sacroiliac joint. Invest Radiol 1975;10(6):608–21. 1201940 10.1097/00004424-197511000-00008

[32] Pialat JB Di Marco L Feydy A Peyron C Porta B Himpens PH Sacroiliac joints imaging in axial spondyloarthritis Vol. 97. Diagnostic and Interventional Imaging. Elsevier Masson SAS; 2016. p. 697–708. 10.1016/j.diii.2016.02.013 27050638

[33] Eshed I Lidar M. MRI findings of the sacroiliac joints in patients with low back pain: Alternative diagnosis to inflammatory sacroiliitis. Isr Med Assoc J 2017 Nov 1;19(11):666–9. 29185277

[34] Jans L Van Praet L Elewaut D Van Den Bosch F Carron P Jaremko JL MRI of the SI joints commonly shows non-inflammatory disease in patients clinically suspected of sacroiliitis. Eur J Radiol 2014 Jan;83(1):179–84. 24168927 10.1016/j.ejrad.2013.10.001

[35] Ehara S El-Khoury GY Bergman RA. The accessory sacroiliac joint: A common anatomic variant. Am J Roentgenol 1988;150(4):857–9. 3258099 10.2214/ajr.150.4.857

[36] Klang E Lidar M Lidar Z Aharoni D Eshed I. Prevalence and awareness of sacroiliac joint alterations on lumbar spine CT in low back pain patients younger than 40 years. Acta radiol 2017 Apr 1;58(4):449–55. 27445315 10.1177/0284185116656490

[37] El Rafei M Badr S Lefebvre G Machuron F Capon B Flipo RM Sacroiliac joints: anatomical variations on MR images. Eur Radiol 2018 Dec 1;28(12):5328–37. 29876707 10.1007/s00330-018-5540-x

[38] Hod N Ashkenazi I Levi Y Fire G Drori M Cohen I Characteristics of skeletal stress fractures in female military recruits of the Israel defense forces on bone scintigraphy. Clin Nucl Med 2006;31(12):742–9. 17117066 10.1097/01.rlu.0000246632.11440.70

[39] Johnson AW Weiss CB Stento K Wheeler DL. Stress fractures of the sacrum. An atypical cause of low back pain in the female athlete. Am J Sports Med 2001;29(4):498–508. 11476393 10.1177/03635465010290042001

[40] Solmaz D Soysal O Ozaksoy D Akar S. Bone marrow edema in the sacroiliac joint due to sacral stress fracture Vol. 19. Journal of Clinical Rheumatology. J Clin Rheumatol. 2013 Aug;19(5):294–5. 10.1097/RHU.0b013e31829d5464 23884188

[41] Abid H Chaabouni S Frikha F Toumi N Souissi B Lahiani D Contribution of imaging in the diagnosis of infectious sacroiliitis: about 19 cases. Pan Afr Med J 2014;17:171. 25120884 10.11604/pamj.2014.17.171.2716 PMC4119445

[42] Slobodin G Rimar D Boulman N Kaly L Rozenbaum M Rosner I Acute sacroiliitis. Clin Rheumatol 2016 Apr;35(4):851–6. 26847855 10.1007/s10067-016-3200-6

[43] Hermet M Minichiello E Flipo RM Dubost JJ Allanore Y Ziza JM Infectious sacroiliitis: A retrospective, multicentre study of 39 adults. BMC Infect Dis 2012 Nov 15;12. 10.1186/1471-2334-12-305 PMC3519695 23153120

[44] Wu MS Chang SS Lee SH Lee CC. Pyogenic sacroiliitis - A comparison between paediatric and adult patients. Rheumatology 2007 Nov;46(11):1684–7. 17901064 10.1093/rheumatology/kem201

[45] Mehanic S Baljic R Mulabdic V Huric-Jusufi I Pinjo F Topalovic-Cetkovic J Osteoarticular manifestations of brucellosis. Med Arh 2012;66(3 Suppl 1):24–6. 22937686 10.5455/medarh.2012.66.s24-s26

[46] Arkun R Mete BD. Musculoskeletal brucellosis. Semin Musculoskelet Radiol 2011;15(5):470–9. 22081282 10.1055/s-0031-1293493

[47] Bozgeyik Z Aglamis S Bozdag PG Denk A. Magnetic resonance imaging findings of musculoskeletal brucellosis. Clin Imaging 2014;38(5):719–23. 24849195 10.1016/j.clinimag.2014.04.007

